# Comparison of Approximate Mathematical Formulas for Optimal Organ Absorbed Dose Estimation in CT Examination of the Abdominopelvic Region

**DOI:** 10.7759/cureus.95533

**Published:** 2025-10-27

**Authors:** Masato Takanashi, Isao Kuroda, Shinji Sugahara, Masataka Hoshina, Masaya Noguchi, Masayoshi Matsushita, Koichi Masuda

**Affiliations:** 1 Department of Radiology and Radiation Oncology, Tokyo Medical University Ibaraki Medical Center, Inashiki-gun, JPN; 2 Department of Urology, Tokyo Medical University Ibaraki Medical Center, Inashiki-gun, JPN

**Keywords:** absorbed dose, computed tomography, dose-length product, dose management software, organ-specific, size-specific dose estimate

## Abstract

Computed tomography (CT) examinations account for a large proportion of all medical exposures. Therefore, various studies on radiation exposure have been conducted. The automatic exposure control system is commonly used in CT examinations, and its characteristics vary from equipment to equipment, which may affect the approximate formulas and coefficients of determination (R^2^). The purpose of this study is to compare the organ-specific absorbed dose approximate formulas for different CT equipment with the literature and to verify the optimal approximate formula.

The subjects were 224 consecutive adult male patients who underwent abdominopelvic CT examinations in 2020, using an Aquilion ONE/PRISM Edition CT equipment (Canon Medical Systems, Inc., Tochigi, Japan). The vertical axis was the absorbed dose to the organs estimated by Monte Carlo simulation using Radimetrics version 3.3 (Bayer Medical Care Inc., Whippany, NJ), a dose management software (DMS). The target organs were the liver, kidney, and colon. The horizontal axis was the dose-length product (DLP) and the size-specific dose estimate (SSDE) calculated by Radimetrics. The approximate formulas analyzed were linear, power, and logarithmic.

All organ-absorbed doses had the highest R^2^ in the logarithmic approximation. By calculating the approximate formula from specimens of one's own facility, one can estimate the absorbed dose for each organ with a high degree of accuracy without using DMS.

## Introduction

Numerous studies exist examining the physical effects of radiation on atomic bomb survivors. While the excess rate of radiation-related cancers increases throughout the study period, new findings indicate that the relative risk decreases with increasing age at exposure, and as mentioned earlier, is highest among those exposed during childhood [1]. Furthermore, as a useful representative value, it has been shown that for individuals exposed at age 30, the risk of solid cancer increases by 47% per 1 sievert by age 70 [1]. Furthermore, radiation exposure from the atomic bomb significantly increased the risk of cancer death in most major organs, including the stomach, lungs, liver, colon, breasts, gallbladder, esophagus, bladder, and ovaries [2]. Computed tomography (CT) examinations account for a large proportion of all medical exposures. Therefore, various studies on radiation exposure have been conducted. In the UK, it was suggested that approximately 0.6% of the cumulative cancer risk up to age 75 may be attributable to diagnostic X-rays. Furthermore, in Japan, which has the world's highest estimated annual exposure frequency, the estimated attributable risk exceeded 3% [3]. The effects of low-dose radiation exposure remain controversial, but Myles et al. have reported that even at a low dose of 5 mSv, there is a potential increase in cancer risk [4]. As these reports demonstrate, managing low-dose radiation exposure in medical settings is critically important. Pearce et al. reported that in children, cumulative doses of approximately 50 mGy from CT scans may nearly triple the risk of leukemia, while doses around 60 mGy may triple the risk of brain tumors [5]. Furthermore, epidemiological studies have demonstrated a clear dose-dependent induction of thyroid cancer, confirming that radiation exposure is the primary cause of thyroid cancer induction [6]. Another report states that the link between radiation exposure and thyroid cancer incidence has been sufficiently established, with the two primary risk factors for thyroid cancer being the amount of radiation delivered to the thyroid and the age at exposure [7]. Numerous other studies have also focused on the absorbed dose per organ [8-13]. However, previous studies have pointed out that despite growing concerns about medical radiation exposure, patient information regarding the risk of radiation-induced cancer remains limited [14]. Additionally, there are reports that patients are unaware of the risk of radiation-induced cancer [15-17]. Therefore, it is important for healthcare professionals, including physicians, to recognize these risks and share information with patients [18,19]. Another report concluded that individual cancer risk estimates must be based on specific information such as exposure circumstances, age at exposure, and absorbed dose to specific tissues. Therefore, it states that doses from medical exposure should be evaluated individually [20,21]. Therefore, determining the absorbed dose for each organ is critically important from the perspective of providing appropriate patient explanations regarding medical radiation exposure. In a previous study, Iriuchijima et al. reported the usefulness of approximate formulas for organ-specific absorbed doses in abdominopelvic CT examinations using a CT equipment manufactured by Siemens Healthcare [22]. However, the automatic exposure control (AEC) system is commonly used in CT examinations, and its characteristics vary from equipment to equipment, which may affect the approximate formulas and coefficients of determination (R^2^) [23,24]. Takanashi et al. reported on the usefulness of conversion factors for accurately estimating effective dose, an indicator for cancer risk assessment [25]. The purpose of this study is to compare the organ-specific absorbed dose approximate formulas for different CT equipment with previous studies and to verify the optimal approximate formula.

## Materials and methods

The subjects were 224 consecutive adult male patients who underwent abdominopelvic CT examinations in 2020, using an Aquilion ONE/PRISM Edition CT equipment (Canon Medical Systems, Inc., Tochigi, Japan) (Table 1). No contrast agent was used in any of the cases. The CT examination protocol used in this study is shown in Table 2. The scanning method used was helical scanning, and the tube voltage was 120 kV. Volume exposure control (EC) was used as the AEC. Volume EC is a 3D mA modulation that automatically modulates the tube current in the XYZ direction. Rotation time was 500 msec, and pitch factor was 0.813. A nominal single collimation width of 0.5 mm was used. Detector rows were 80, and the setting standard deviation was 15.

**Table 1:** Number of samples and age statistics.

Number of samples	224
Age (mean ± SD)	63 ± 18

**Table 2:** CT examination protocol used in this study.

Scan type	Helical
Tube voltage (kV)	120
Volume exposure control (XYZ tube current modulation)	ON
Rotation time (msec)	500
Pitch factor	0.813
Nominal single collimation width (mm)	0.5
Number of detector rows	80
Nominal total collimation width (mm)	40
Setting standard deviation	15

**Figure 1:**
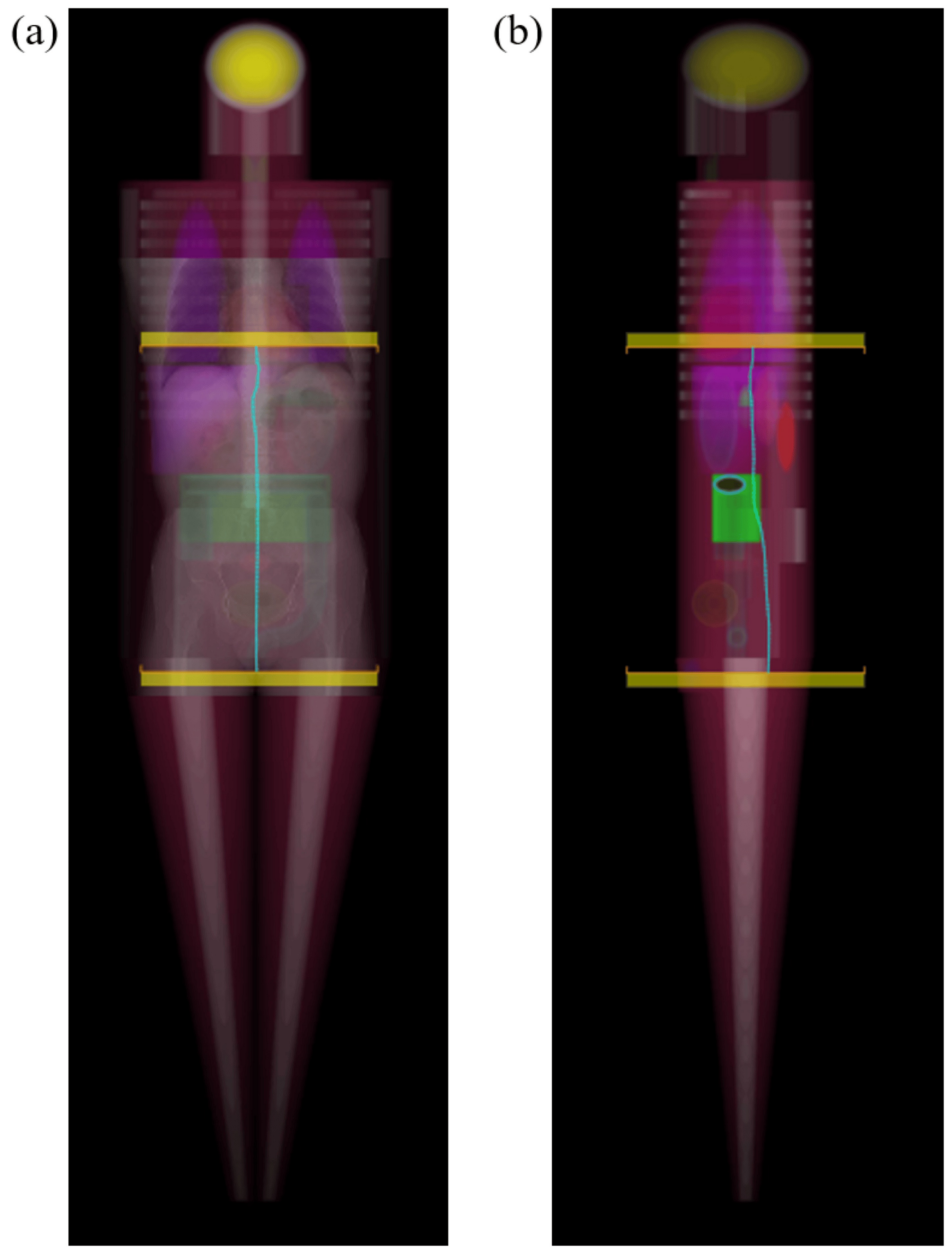
Image projected onto the phantom from the scan area. The figure shows an image of the scan area projected onto the phantom. Monte Carlo simulation is used to estimate the organ absorbed dose. Panel (a) shows an image of the phantom viewed from the front. Panel (b) shows an image of the phantom viewed from the side. There are 54 different mathematical phantoms used for the estimation, which is based on the gender, age, and body size, and the phantom that most closely resembles the patient is selected. X-ray output information is collected from the Digital Imaging and Communication in Medicine (DICOM) Radiation Dose Structured Report (RDSR), dose sheet, and axial image header information. The imaging area used in the Monte Carlo simulation is determined from the imaging area information in the DICOM tag or RDSR. The scan area is obtained from the DICOM RDSR. Then, the position is determined so that the center of the scan area corresponds to the center of the imaging site, and the organ doses and effective doses are calculated.

Arm hanging positions and metal implants were excluded from the specimens. The vertical axis is the absorbed dose to the organs estimated by Monte Carlo simulation of Radimetrics version 3.3 (Bayer Medical Care Inc., Whippany, NJ), a dose management software (DMS). There are 54 different mathematical phantoms used for the estimation, which is based on the gender, age, and body size, and the phantom that most closely resembles the patient is selected. Radimetrics uses gender, age, and phantom size as criteria for selecting the optimal mathematical phantom. For phantom size, weight, water equivalent diameter, and effective diameter are referenced. X-ray output information is collected from the Digital Imaging and Communication in Medicine (DICOM) Radiation Dose Structured Report (RDSR), dose sheet, and axial image header information [25,26]. Figure 1 shows the image projected onto the phantom in the scan area. Figure 1A shows an image of the phantom viewed from the front. Figure 1B shows an image of the phantom viewed from the side. The imaging area used in the Monte Carlo simulation is determined from the imaging area information in the DICOM tag or RDSR. The scan area is obtained from the DICOM RDSR. Then, the position is determined so that the center of the scan area corresponds to the center of the imaging site, and the organ doses and effective doses are calculated. The target organs were the liver, kidney, and colon.

The horizontal axis was the dose-length product (DLP) and the size-specific dose estimate (SSDE) calculated by Radimetrics [27-29]. We analyzed the formula giving the highest coefficient of determination (R^2^) value. The approximate formulas analyzed were linear, power, and logarithmic. In this study, the approximation formula yielding the highest R² was defined as optimal. The SSDE in this study was the mean value obtained from each slice. Microsoft Office Excel 2019 (Microsoft Corporation, Redmond, WA) was used for the analysis.

This study utilized various patient-specific mathematical phantoms from Radimetrics to estimate organ absorbed doses for individual patients. Regarding the accuracy of Radimetrics estimates, Iriuchijima et al. concluded that the relative difference between measured and simulated organ doses is comparable to that of Fujii et al. [30] and is useful for assessing organ doses in individual patients [31]. The results of previous studies are similar to those reported in the American Association of Physicists in Medicine (AAPM) 246 report [32], and the estimation accuracy of Radimetrics is considered comparable to that of existing software [30-32].

## Results

**Figure 2:**
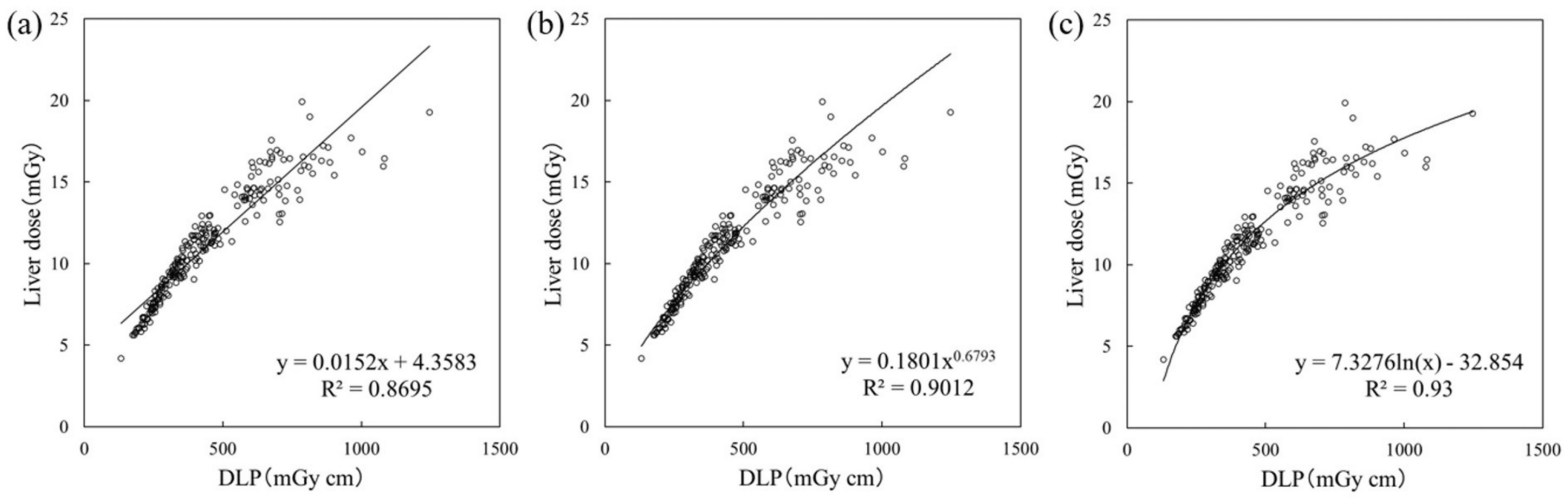
Formula for organ absorbed dose (liver). DLP: dose-length product; R^2^: coefficients of determination.

Figure 2 shows the formula and R² when the vertical axis is set to the organ absorbed dose to the liver, and the horizontal axis is set to DLP. Figure 2A shows linear regression, yielding an R² value of 0.8695. Figure 2B shows power regression, yielding an R² value of 0.9012. Figure 2C shows logarithmic regression, yielding an R² value of 0.9300. From this, logarithmic regression yielded the maximum R² value.

**Figure 3:**
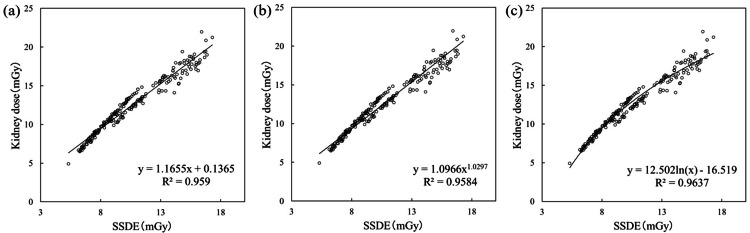
Formula for organ absorbed dose (kidney). SSDE: size-specific dose estimate; R^2^: coefficients of determination.

Figure 3 shows the formula and R² when the vertical axis is set to the organ absorbed dose to the kidney, and the horizontal axis is set to SSDE. Figure 3A shows linear regression, yielding an R² value of 0.9590. Figure 3B shows power regression, yielding an R² value of 0.9584. Figure 3C shows logarithmic regression, yielding an R² value of 0.9637. From this, logarithmic regression yielded the maximum R² value.

**Figure 4:**
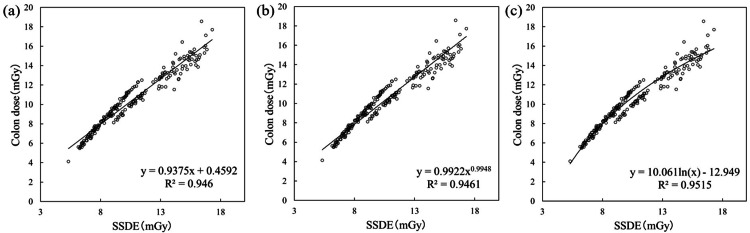
Formula for organ absorbed dose (colon). SSDE: size-specific dose estimate; R^2^: coefficients of determination.

Figure 4 shows the formula and R² when the vertical axis is set to the organ absorbed dose of the colon, and the horizontal axis is set to SSDE. Figure 4A shows linear regression, yielding an R² value of 0.9460. Figure 4B shows power regression, yielding an R² value of 0.9461. Figure 4C shows logarithmic regression, yielding an R² value of 0.9515. From this, logarithmic regression yielded the maximum R² value.

## Discussion

Regarding organ dose estimation methods using approximate formulas, Turner et al. developed an estimation method using volume CT dose index (CTDI_vol_) for both pediatric and adult patients [33]. However, Turner et al.'s method was evaluated without using AEC, making it unsuitable for modern clinical practice where AEC is widely adopted. The studies by Iriuchijima et al. and our own study evaluated methods using AEC, aligning with current examination practices.

Table 3 shows the results of this study compared with those of Iriuchijima et al. [22]. For all organs examined, the vertical axis and approximate formula with the highest R^2^ were the same as those of Iriuchijima et al. All organ-absorbed doses had the highest R^2^ in the logarithmic approximation. Liver dose = 7.33ln (DLP) -32.9 (R^2 ^= 0.930), kidney dose = 12.5ln (SSDE) -16.5 (R^2 ^= 0.964), and colon dose = 10.1ln (SSDE) -12.9 (R^2 ^= 0.952).

**Table 3:** Results of this study compared with those of Iriuchijima et al. For all organs examined, the vertical axis and approximate formula with the highest R^2^ were the same as those of Iriuchijima et al. [22]. All organ-absorbed doses had the highest R^2^ in the logarithmic approximation. Liver dose = 7.33ln (DLP) -32.9 (R^2 ^= 0.930), kidney dose = 12.5ln (SSDE) -16.5 (R^2 ^= 0.964), colon dose = 10.1ln (SSDE) -12.9 (R^2 ^= 0.952). DLP: dose-length product; SSDE: size-specific dose estimate; R2: coefficients of determination.

Organ	CT index	Iriuchijima et al. [22]		Our study	
		Formula (y: organ dose, x: index)	R^2^	Formula (y: organ dose, x: index)	R^2^
Liver	DLP	y = 12.6ln (x)-61.7	0.871	y = 7.33ln (x)-32.9	0.930
Kidney	SSDE	y = 22.2ln (x)-42.4	0.891	y = 12.5ln (x)-16.5	0.964
Colon	SSDE	y = 18.0ln (x)-33.5	0.876	y = 10.1ln (x)-12.9	0.952

Iriuchijima et al. reported that selecting the optimal approximation formula for each organ is necessary to estimate organ doses using dose indices [22]. However, comparing the research by Iriuchijima et al. with our own revealed that the slopes and intercepts of the formulas differ. In other words, when creating formulas for CT dose indices and organ absorbed doses from past data, the trends of the optimal formulas are similar, but the slope and intercept must be determined for each facility. Furthermore, Iriuchijima et al. evaluated the scan area from the chest to the pelvic region and reported that CTDI_vol_ showed high R^2^ values in the breast, DLP in the liver, and SSDE in the lungs, heart, kidneys, and colon. They attributed the higher R^2^ for DLP in the liver alone among abdominal organs to the large density variation near the diaphragm, spanning from the lung fields to the abdominal parenchymal organs. Iriuchijima et al. suggested this caused the correlation to deteriorate for SSDE. This study verified the dose index deemed optimal by Iriuchijima et al. for the horizontal axis; however, future research should also verify the results when the horizontal axis is replaced with other indices.

Various prior studies have indicated that organ absorbed doses are necessary for a detailed assessment of the physical effects of radiation [5-13]. Furthermore, communication errors regarding explanations of medical radiation exposure in clinical settings have been identified [14-17]. Going forward, as similar research reports are collected, it is anticipated that the characteristics of approximation formulas for each manufacturer and target scan area will become clearer. This will enable more specific explanations to patients regarding medical radiation exposure, thereby contributing to the provision of reassuring medical care.

This study verified whether the approximate formula for estimating organ absorbed dose in the abdominopelvic region remains consistent across different equipment from Siemens Healthcare and Canon Medical Systems. In the future, we aim to verify whether the characteristics obtained in this study are consistent across equipment from other manufacturers. Furthermore, we intend to investigate formulas for organ absorbed dose targeting other scan regions, such as the head and chest. Furthermore, this study validated the approximation formula using only male subjects. We aim to expand its applicability to include women and children in future work.

As a limitation, AEC is commonly used in CT examinations, and since its characteristics vary by equipment, it may affect approximation formulas and R².

## Conclusions

In this study, comparing organ-specific absorbed dose approximation formulas with the literature revealed that the trend toward optimal approximation formulas is similar, even across different CT equipment. We plan to expand the number of target devices for further verification. By calculating the approximate formula from specimens of one's own facility, one can estimate the absorbed dose for each organ with a high degree of accuracy without using DMS.
